# Cushing syndrome secondary to ectopic adrenocorticotropic hormone secretion from a Meckel diverticulum neuroendocrine tumor: case report

**DOI:** 10.1186/s12902-015-0070-x

**Published:** 2015-11-26

**Authors:** Diana Loreta Paun, Lavinia Vija, Emilia Stan, Alexandra Banica, Elena Bobeica, Dana Terzea, Catalina Poiana, Corin Badiu, Sorin Paun

**Affiliations:** “C.I. Parhon” National Institute of Endocrinology, 34-36, Aviatorilor Boulevard, sector 1, 011863 Bucharest, Romania; “Carol Davila” University of Medicine and Pharmacy, Bucharest, Romania; Braila Department Hospital, Braila, Bucharest Romania; General Surgery Department, Bucharest Emergency Hospital, Bucharest, Romania

**Keywords:** Ectopic Cushing’s syndrome, Ectopic ACTH secretion, Meckel diverticulum, Neuroendocrine tumor, Hipokalaemia

## Abstract

**Background:**

Ectopic production of adrenocorticotropic hormone (ACTH) by neuroendocrine tumours (NET) is a rare condition, occult presentations often hampering the diagnosis. Although NET are relatively frequent in the ileon and Meckel diverticulum, we describe the first Cushing’s syndrome due to ectopic adrenocorticotropic syndrome (CS-EAS) arising from a Meckel diverticulum.

**Case presentation:**

A 44-year-old man was admitted with recent onset of diabetes, myopathy, edema and hypokalemic metabolic alkalosis consistent with Cushing’s syndrome. Both basal and dynamic laboratory evaluation suggested CS-EAS. Laboratory testing also showed high serum levels of chromogranin A (CgA) and urinary 5-hydroxyindoleacetic acid (5HIAA). Pituitary and neck/thorax/abdomen/pelvis imaging proved to be normal, while somatostatin analogue (^99m^Tc-HYNIC-TOC) scintigraphy revealed increased focalized ileum uptake on the right iliac fossa. Pre-operative ketoconazole and sandostatin treatment controlled the hypercortisolism within a month. Pathological analysis of the resected submucosal 1.8 cm tumour of the Meckel diverticulum and a metastatic local lymph node confirmed a well differentiated neuroendocrine tumour (grade I), whereas immunohistochemistry was positive for ACTH, chromogranin A and synaptophysin. Post-operative clinical and biochemical resolution of Cushing’s syndrome was followed by normalization of both CgA and 5HIAA, which were maintained at the 6 month follow-up.

**Conclusion:**

The identification, characterization and follow-up of this rare cause of ectopic ACTH secretion is important in order to assess the long-term prognostic and management.

## Background

Cushing syndrome (CS), secondary to ectopic adrenocorticotropic hormone secretion (CS-EAS), was reported initially in 1928, while the link between CS and a nonpituitary tumor as a source of EAS was established in 1962 [1], was recently estimated to constitute up to 18 % of all causes of CS [2–8].

Among the variety of tumours, mostly of neuroendocrine origin, associated with CS-EAS [2–4, 7, 8], gastroenteropancreatic tumors (GEP NET) represent 9–17 % [2, 3, 5, 9, 10]. Whereas NET arising from Meckel diverticulum are very rare (more than 120 cases described in the literature [11–15], harbouring particular features among ileal neuroendocrine tumours, from our knowledge, CS-EAS from a Meckel diverticulum was not previously described.

We present the first case of an ectopic adrenocorticotropic hormone secretion, from a Meckel diverticulum neuroendocrine tumor.

## Case presentation

A 44-year-old Caucasian man was admitted in June 2014 to the hospital with recent (5 months) onset of ankle oedema, high blood glucose and low potassium levels (1.9 mmol/L). Physical examination showed centripetal distribution of the fat tissue with 10 kg (15 %) weight loss (BMI 25 kg/m^2^) over 5 months, moon face, buffalo hump, facial rash, dispersed small bruises on the trunk, generalized muscle weakness and muscle hypotrophy of the lower limbs. The remaining physical examination was normal, including blood pressure levels.

Increased ACTH and cortisol levels, high blood glucose, low potassium and metabolic alkalosis (Table 1) pointed to an ACTH-dependent Cushing syndrome.

**Table 1:** Laboratory data

Variable	On admission	After surgery	Reference range, Adults
Sodium	145	140	135–145 mmol/L
Potassium	1.9	3.7	3.5–4.5 mmol/L
HCO_3_^−^	33	26	24–32 mmol/L
Blood glucose	124	82	70–100 mg/dl
HbA1c	8.2		<6 %
Plasma cortisol	>61.4	8	6.2–19.4 μg/dl
Plasma cortisol After 1 mg DXM O/N	>61.4		6.2–19.4 μg/dl
After 8 mg 2-d DXM test	>61.4		6.2–19.4 μg/dl
24 h-urinary cortisol	736.8		58–403 μg/24 h
After 8 mg 2-d DXM test	13300		58–403 μg/24 h
ACTH	487	18	3–66 pg/ml
Chromogranin A	229	42	20–125 ng/ml
5HIAA	17		1–10 mg/24 h
NSE	33	27	0–12 ng/ml
Serotonin	188		40–400 ng/ml

The high-dose dexamethasone suppression test (2 mg of dexamethasone every 6 h during 48 h starting at 8 a.m.) showed no cortisol suppression confirming the CS-EAS diagnosis (Table 1). Tumour markers determination showed increased levels of CgA, neuronal specific enolase (NSE), serotonin and 5-hydroxyindoleacetic acid (5-HIAA) (Table 1), all reinforcing an EAS.

**Fig. 1:**
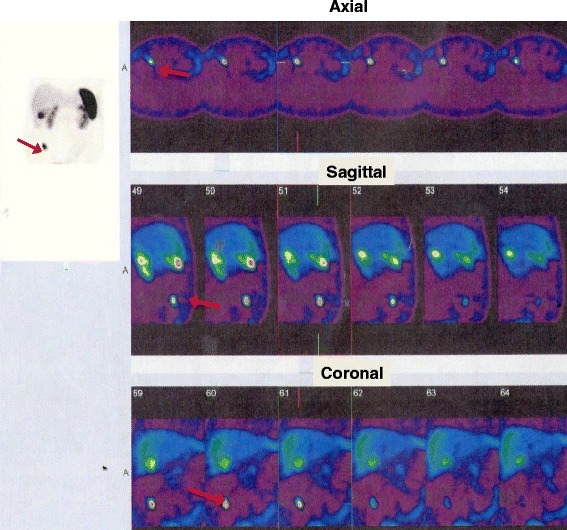
Whole body scan with somatostatin analogue, ^99m^Tc-HYNIC-TOC (Tektrotyd), showing a single nodular region in the inferior third of the right iliac fossa (red arrow)

Pituitary MRI revealed a pituitary 4 mm adenoma. Thorax, abdomen and pelvis CT did not reveal any tumour, (data not shown) while adrenal glands were hyperplastic. In this context, a whole body scintigraphy using ^99m^Tc-HYNIC-Tyr^3^-Octreotide (Tektrotyd), previously described as a useful method for diagnosis, staging and follow up of the patients with NETs [16], was performed and revealed increased focal ileum uptake in the right iliac fossa (Fig. 1).

The osteodensitometry showed vertebral osteoporosis with a T-score of −2.9 and Z-score of −2.6, consistent with secondary osteoporosis.

**Fig. 2:**
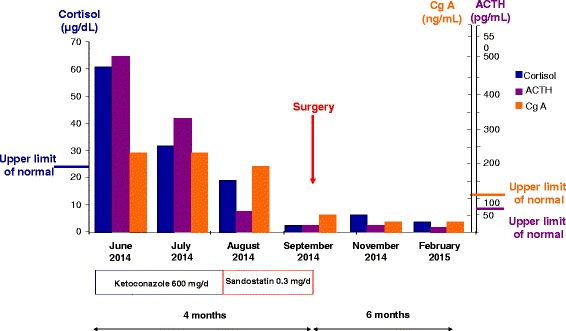
Histogram of plasmatic cortisol, ACTH and CgA levels evolution during medical treatment (with ketoconazole and sandostatin), as well as after the surgical resection of the Meckel diverticulum tumour and during 6 month follow-up. ACTH-adrenocorticotropic hormone; CgA-chromogranin A

After the EAS diagnosis, both ketoconazole (400 mg/d) and sandostatin (100 μgx3/d) treatment alleviated hypercortisolism within a month (Fig 2). For the secondary diabetes mellitus, metformin (850 mgx2/d) was prescribed and a good glycaemic control was achieved. Secondary osteoporosis was treated with calcium and vitamin D (900 mg of calcium citrate with 600 IU of choleclaciferol daily).

**Fig. 3:**
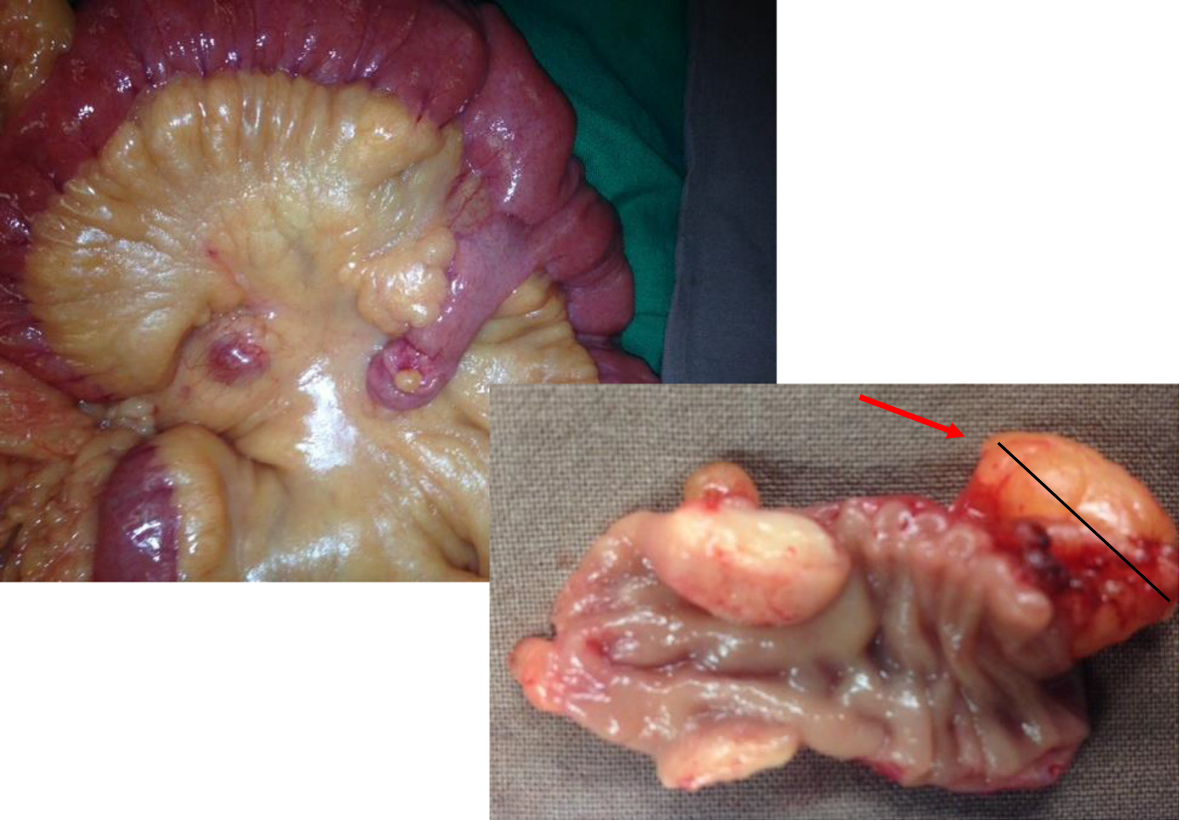
Long Meckel diverticulum with 1.8/1 cm submucosal nodular tumor (red arrow)

**Fig. 4:**
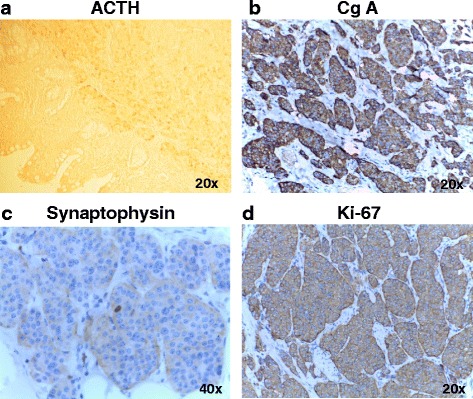
Tumoral positive immunostaining for ACTH (**a**), CgA (**b**), synaptophysin (**c**) and Ki-67(**d**) 20x magnification, for **a**,**b**,**d**; 40x magnification for **c**. ACTH- adrenocorticotropic hormone; CgA-chromogranin A

Laparoscopic abdominal exploration revealed a 12 cm long Meckel’s diverticulum with an irregular shape and a 1.8 cm nodular tumor mass (Fig. 3). Diverticulectomy with regional lymph node resection was elected, while intraoperative small bowel inspection revealed no luminal damage. Pathological analysis of the resected submucosal 1.8 cm tumour of the Meckel diverticulum confirmed a well differentiated neuro-endocrine tumour (grade I), whereas immunohistochemistry was positive for ACTH, chromogranin A and synaptophysin; Ki-67 score was < 1 % (Fig. 4). There was one local metastatic lymph node.

One month as well as 6 months post-operatively, plasmatic cortisol, ACTH and tumoral markers (CgA, 5HIAA) were within normal limits (Fig. 2, Table 1).

## Conclusions

Neuroendocrine tumors in Meckel’s diverticulum resemble to ileal NET in their biological behavior more than to appendiceal NET. It was recently reported that 77 % of Meckel diverticulum tumors present histological characteristics for a neuroendocrine origin, but when compared to ileal tumors, the risk of cancer in the Meckel diverticulum was 70 times higher than any other ileal site [17]. In a review of 104 Meckel’s cases of NET, Nies et al. [13] found an incidence 2.5 greater in men, a frequent localization on the tip of the diverticulum and an exceptional association with metastatic carcinoid syndrome of less than 2 cm. The presented case had a less than 2 cm well differentiated NET with local lymph node extension. Jejuno-ileal NET represent 30**–**50 % of all small bowel neoplasms, with 72 % survival rates at 5 years for tumours with loco-regional spread [14, 18]. Along with this line, we expect that our patient might follow the same clinical course, or even better, as the histological type corresponded to a well differentiated tumor G1 with Ki67 < 1 %.

Ectopic ACTH syndrome produced by a NET from the gastrointestinal tract is extremely rare and only described in isolated case reports. While only five cases of CS-EAS form appendicular NET were reported in the literature [2, 19–22], from our knowledge we present the first case of CS-EAS from a Meckel diverticulum well differentiated NET.

Clinical features of ectopic ACTH syndrome depend on the source of production and rate of ACTH synthesis. Characteristically, these patients have severe hypercortisolemia leading to low serum potassium levels, diabetes, generalized infections, hypertension, and psychosis. Whether rapidly growing tumours, such as small cell lung cancer typically produce profound and sudden onset of symptoms, well defined Cushingoid body habitus changes are noticed in slower growing tumours such as jejuno-ileal NET, as was demonstrated in this patient.

The current case is the first report of a Meckel diverticulum NET-producing ACTH presenting clinically as Cushing’s syndrome. The diagnosis was assisted by a positive somatostatin analogue whole body scintigraphy.

Localization of EAS tumours is often challenging as occult tumours have been described in 12–19 % of adult patients [2–5, 23]. Modern cross-sectional imaging (CT and/or MRI of the neck/thorax/abdomen/pelvis) are recommended as first line investigations [24]. When these are negative, functional imaging techniques in adults (somatostatin analogues scintigraphy, coupled with SPECT/CT,^18^F-DOPA-PET-CT or ^68^Ga-labelled peptides-PET-CT) brought promising results in the localization of jejuno-ileal NET [23, 25, 26].

There have been described only two cases of Meckel diverticulum carcinoma with uptake of FDOPA-PET, FDG-PET and somatostatin receptor scintigraphy [26].

Metabolic PET scanning using 18-fluorodeoxyglucose (FDG) was not usually recommended since it has a low sensitivity for jejuno-ileal NET, due to low metabolic activity in G1 tumors. Indeed, the sensitivity of CT and combined somatostatin analogues scintigraphy in the localization CS-EAS was found to be higher (53 %) than that of MRI (37 %) or FDG-PET (35 %) [27].

In the presented case, the somatostatin analogue scintigraphy allowed the localization of the EAS tumour source. Surgical resection was the definitive and optimal management of this rare condition and led to a complete resolution of symptoms.

Further studies on more cases would be needed in order to evaluate the best follow-up procedure, as well as the long term progression free survival and outcome for patients with this rare localization of ileal neuro-endocrine tumor.

## Consent

Written informed consent was obtained from the patient. A copy of the written consents is available upon request for review by the Journal Editor.
